# Advances in targeted degradation of endogenous proteins

**DOI:** 10.1007/s00018-019-03112-6

**Published:** 2019-04-27

**Authors:** Sascha Röth, Luke J. Fulcher, Gopal P. Sapkota

**Affiliations:** MRC Protein Phosphorylation and Ubiquitylation Unit, School of Life Sciences, University of Dundee, Dow Street, Dundee, DD1 5EH UK

**Keywords:** Nanobody, Monobody, Ubiquitin, Proteasome, Auxin, Affinity-directed protein missile, VHL, CRBN, AiD, HALO, FKBP12, Thalidomide, Proteolysis targeting chimera, PROTAC

## Abstract

Protein silencing is often employed as a means to aid investigations in protein function and is increasingly desired as a therapeutic approach. Several types of protein silencing methodologies have been developed, including targeting the encoding genes, transcripts, the process of translation or the protein directly. Despite these advances, most silencing systems suffer from limitations. Silencing protein expression through genetic ablation, for example by CRISPR/Cas9 genome editing, is irreversible, time consuming and not always feasible. Similarly, RNA interference approaches warrant prolonged treatments, can lead to incomplete protein depletion and are often associated with off-target effects. Targeted proteolysis has the potential to overcome some of these limitations. The field of targeted proteolysis has witnessed the emergence of many methodologies aimed at targeting specific proteins for degradation in a spatio-temporal manner. In this review, we provide an appraisal of the different targeted proteolytic systems and discuss their applications in understanding protein function, as well as their potential in therapeutics.

## Introduction

Understanding the inherent function of a protein is a cornerstone in life sciences. Many biochemical and biophysical properties of proteins can be ascertained in vitro; however, a key drawback of these approaches is the absence of the cellular context. In cellulo research on the function of a protein of interest (POI) often relies on its deletion or depletion from cells to screen for changes in, for example, phenotype, signalling or gene expression patterns. Two current routine approaches used to achieve POI deletion or depletion are genome editing, for example by using CRISPR/Cas9-mediated gene knockout, and RNA interference (RNAi), respectively.

**Fig. 1:**
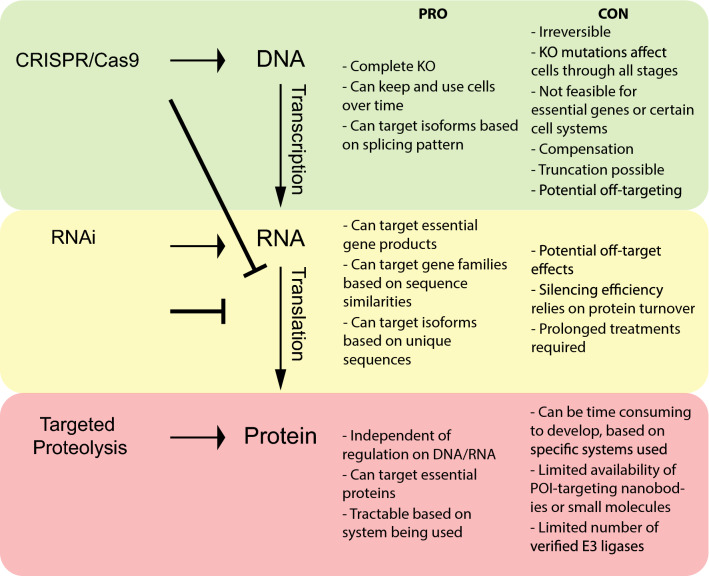
Comparison of protein silencing tools and their advantages (PRO) and disadvantages (CON). Top: CRISPR/Cas9 genome editing results in mutations of target gene sequence, typically through deletion of the start ATG codon, and introduction of early stop codons or frame shifts. While transcripts are still produced, translation is blocked. Middle: RNAi approaches result in cleavage of the mRNA transcripts. This in turn effectively blocks translation of the protein. Bottom: targeted proteolysis: While both transcription and translation remain unaffected, through mechanisms described for the different proteolytic methods, the target protein is directly marked for proteasomal degradation

With recent advances in CRISPR/Cas9 genome editing, targeted knockout of genes in cells, tissues and whole organisms has been widely utilized to assess protein function. CRISPR/Cas9 is a bacterial and archaeal defence mechanism against viral infections, which acts to cleave foreign DNA or RNA from invading pathogens, thereby limiting infection [1]. Back in 2012, this system was adapted as a powerful genome editing tool [2] which could be utilized to either knockout a target gene, or introduce new DNA sequences into the genome in eukaryotic cells (Fig. 1, top). While CRISPR/Cas9 has since become an easy, cost-effective method for genome editing, it cannot be used for all targets, as single cell clones of essential gene knockouts will not survive the clonal selection process. Additionally, CRISPR/Cas9 genome editing usually does not interfere with transcription, but relies on the deletion of the start codon or introduction of nonsense mutations. However, mutagenesis by CRISPR can result in exon skipping, generating a different start codon, resulting in truncations of the protein still being translated [3]. Even in the case of a true gene knockout, however, the time required for a single cell clone to propagate might be sufficient for cells to establish genetic compensation, thereby altering expression patterns of related proteins to make up for the loss of one protein [4]. Because gene knockouts are irreversible, the homeostatic changes in knockout cell clones might be very different from parental cells. Therefore, any interpretation of changes due to the loss of a POI relies on the ability to rescue phenotypes by restoring the POI expression. This approach is not always feasible and often relies on the use of artificial and constitutive promoters to drive protein expression.

RNAi-mediated depletion of a target POI transcript is a potential way to circumvent some of the problems of CRISPR/Cas9 genome editing highlighted above, as this technique is performed in a much shorter time frame than that required for a genetic knockout. In metazoan cells, RNAi approaches include short interfering RNAs (siRNAs), microRNAs (miRNAs) and short hairpin RNAs (shRNA) [5]. In all of these cases, the RNAi machinery triggers the activation of the RNAi-induced silencing complex (RISC) and, depending on sequence complementarity, results in either degradation of the target RNA or inhibition of its translation (Fig. 1, middle) [6], leading to post-transcriptional gene silencing. While these methods enable the opportunity to target transcripts of essential genes, they ultimately rely on the turnover of the target protein for efficient reduction in target protein levels. For silencing of most POIs, RNAi is usually effective only after 48–72 h of treatment. However, for those proteins that have very slow turnover rates that surpass this time frame [7], RNAi approaches will not yield the desired knockdown of target transcripts. Another common issue associated with RNAi appears to be off-target silencing of transcripts based on partial sequence complementarity [8], despite great efforts going into the optimization of siRNA sequence design or delivery to minimize potential off-target effects [9].

An alternative approach, circumventing the challenges of protein silencing through genomic alterations or transcript inhibition, is the direct targeting of proteins for destruction. Targeted proteolysis is desirable both in therapeutic applications and in basic research. To date, such proteolytic approaches have relied on the cells’ built-in protein recycling centre, known as the ubiquitin–proteasome system (UPS). In short, proteins are marked for destruction through the covalent attachment of the small protein ubiquitin onto primarily lysine residues on the target protein [10, 11]. The first step of the reaction is mediated by the E1 ubiquitin-activating enzyme that activates the ubiquitin monomer. Subsequently, the activated ubiquitin is passed on to the E2 ubiquitin-conjugating enzyme, before ultimately being attached to the target protein through the action of an E3 ubiquitin ligase. The ubiquitin monomers can be further ubiquitinated to form chains, with distinct chain types linked to specific biological processes [12–14]. For example, and of relevance here, if ubiquitin molecules added to the target protein form a lysine 48-linked chain, the ubiquitinated protein is marked for proteasomal degradation [12, 13]. The proteasome itself is a cylindrical multimeric protein complex composed of a 20S core particle consisting of structural alpha subunits, as well as catalytic beta subunits that possess the protease activity [15–17]. At either end of the 20S core particle is a 19S regulatory particle. This regulatory component acts as a gatekeeper to regulate entry of target proteins into the catalytic 20S core, thereby controlling proteolysis [15–17].

The possibility of using the native UPS to selectively degrade POIs for which genes cannot be knocked out genetically or silenced by RNAi is an attractive alternative approach (Fig. 1, bottom). The field of targeted proteolysis is beginning to rapidly gather pace and provides many ways to remove target proteins independently of genomic or transcriptional modulation. All targeted proteolysis approaches rely on the concept of recruiting the POI to an E3 ligase for ubiquitination and subsequent degradation. The first step of targeted proteolysis requires a robust system for target recognition. This can be achieved through a POI-specific high-affinity binder, e.g. a camelid derived nanobody [18, 19] or a synthetic small molecule [20]. In turn, these POI-targeting elements can be interlinked with either the proteolytic UPS-inducing E3 ligase element (often an E3 ligase, its catalytic domain or a substrate receptor of an E3 ligase complex) or with a small molecule targeting the E3 ligase [20, 21]. Alternatively, degradation of the POI can be achieved through fusion of the POI to a degron sequence [22, 23]. In terms of POI-targeting elements, the advances in both small molecules and engineered polypeptides means that many approaches now exist for targeted proteolysis of intracellular POIs. In the following sections, we present several of these systems, highlighting their benefits and limitations.

## Auxin-inducible degron (AiD)

**Fig. 2:**
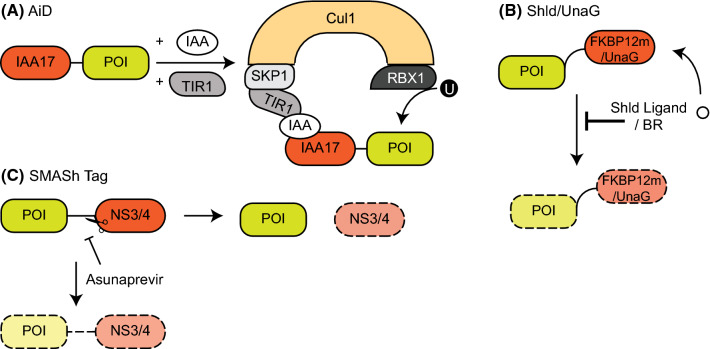
An overview of the proteolytic approaches using degrons. **a** Auxin-inducible degron (AiD): the POI is fused to an IAA17 degron. Expression of TIR1 and addition of auxin (IAA) results in the recruitment of the IAA17-fused POI to TIR1, which in turn is recruited to the SKP1–Cul1–RBX1 complex, resulting in POI ubiquitination by RBX1. **b** Shld and UnaG degrons: in the absence of the stabilizing Shld ligand or bilirubin (BR), the POI is degraded when fused to the destabilizing FKBP12 or UnaG mutants, respectively. Addition of the respective compound stabilizes the fusion protein. **c** SMASh-Tag: the POI is fused to NS3/4 through an NS3 cleavage site. NS3 is cleaved from the POI and degraded, while the native POI is stable (right arrow). Upon addition of the NS3 inhibitor asunaprevir, NS3 cleavage is inhibited and the entire fusion protein is degraded (down arrow)

Plant cell signalling relies on phytohormones like auxin (AUX). Upon exposure to hormones of the auxin family, such as indole-3-acetic acid (IAA), the AUX/IAA family of transcriptional repressor proteins are rapidly degraded through a specific form of the SCF (SKP–cullin–F-Box)-RING E3 Ligase system [24, 25]. Auxin hormones bind to the F-box transport inhibitor response 1 (TIR1) protein and, in doing so, promote the association between the SCF-TIR1 E3 ligase complex with AUX/IAA transcriptional repressors, ultimately leading to the ubiquitination and degradation of the AUX/IAA transcriptional repressors [24, 25]. Whilst other eukaryotes lack this auxin-inducible response, the SCF degradation pathway is conserved, thereby raising the tantalizing possibility that the auxin-inducible degradation pathway could be transferred into other eukaryotic cells. The Kanemaki lab demonstrated this possibility with rapid, reversible degradation of essential POIs in cell lines derived from various organisms including chicken, mouse, yeast, and humans [24]. They achieved this through fusion of an IAA17 degron sequence (AID) to the POI and overexpression of TIR1 in target cells. Rapid POI degradation was observed following IAA treatment (Fig. 2a) [24]. They named this system the auxin-inducible degron [(AiD) with a lowercase i to differentiate it from the AID degron sequence]. As it is generally believed that auxin is only active in plants, the off-target effects of auxin treatment are thought to be minimal. However, IAA oxidation through the action of eukaryotic peroxidases has been reported to be toxic at high doses [26], highlighting the need to test auxin sensitivity in the target cell line before utilizing this system. Ensuring there are no off-target effects of auxin in cells is critical, as relatively large amounts of IAA are required to induce POI degradation in cells [21, 24]. Current efforts in engineering and improving this system resulted in a higher-affinity auxin–TIR1 pair [27]. Future efforts aimed at implementing this “super strong” auxin–TIR1 into the AiD system may allow for lower drug treatments and further improve degradation kinetics.

One of the key limitations of the AiD system is that it cannot be used to explore the effects of endogenous POI degradation without first inserting the IAA degron sequence at the POI locus. This has not been such an impediment in yeast, with demonstrations of robust and rapid AID-mutant generation [24]. Furthermore, as the AiD system does not require temperature modulation in yeast, in contrast to that required for temperature-sensitive degrons [28], it bypasses any off-target effects associated with the temperature adjustment process. However, in metazoan cells the AiD system is more challenging to integrate, owing to the much less efficient homologous recombination compared to yeast.

With advances in CRISPR/Cas9 gene editing technologies, the AID degron sequence was successfully knocked into the genes encoding the cohesin and cytoplasmic dynein proteins in human colorectal cancer HCT116 cells. Following TIR1 over-expression, IAA-responsive cohesin and dynein degradation were demonstrated [21]. Similar results were also observed in mouse embryonic fibroblasts [21]. Whilst AiD presents a powerful tool to achieve POI degradation in cells [21, 24], inserting a non-fluorescent AID tag homozygously into the POI locus by genome editing still remains a huge challenge. A recent study sought to overcome this by tagging POIs with green fluorescent protein (GFP) and modifying the AiD system with an AID–anti-GFP nanobody fusion. In this case, endogenously GFP-tagged proteins in both human cells and zebrafish could be degraded in an auxin-inducible manner, when co-expressing the AID–anti-GFP nanobody fusion as well as TIR1 [29]. With any AiD system, potential off-target effects of over-expressing the TIR1 protein in non-plant cells need to be delineated.

## Conditionally-stable FKBP12 and UnaG tag degrons

Some recent proteolytic advances have centred around the FKBP12–rapamycin–mTORC1 complex. Rapamycin, a highly-studied compound originally isolated from soil samples on Easter Island (Rapa Nui) [30], exerts its biological effects through simultaneously binding FKBP12 and the FRB domain of mTOR, and subsequently inhibiting the master protein kinase complex mTORC1. Given rapamycin’s propensity to bind the FRB domain of mTOR, researchers employed a “bump-and-hole” strategy to generate rapamycin-like molecules that bind poorly to the wild-type FRB domain of mTOR, but maintain strong binding against an engineered form of the FRB domain. For example, the rapamycin-like molecule MaRap was found to bind poorly to wild-type FRB, but very strongly to a mutant of FRB (FRB*) [31], and fusion of this mutant to the kinase GSK-3β resulted in conditional extranuclear localization of GSK-3β [23, 32]. Additionally, in the absence of the MaRap ligand, this FRB*–GSK-3β fusion protein was much less stable than the wild-type FRB–GSK-3β fusion [23, 32]. This instability of the FRB*–GSK-3β fusion protein could be rescued upon MaRap treatment [32]. Thus, the idea that the FKBP12–rapamycin–FRB complex could be exploited for conditionally stable fusion proteins was born [23].

This concept was improved by creating a system that would allow inducible control of protein stability, by fusing mutants of the FKBP12 protein that mediate constitutive and rapid degradation following their expression in mammalian cells [23]. Following administration of a small molecule, Shld1, which binds the mutant FKBP12, the fusion protein is stabilized and therefore free to perform its biological role(s) (Fig. 2b) [23]. This Shld1-dependent stability was demonstrated in various cell lines, including those derived from humans [23], which opens up the possibility of using this system endogenously through CRISPR/Cas9 mediated gene editing of the POI. While this “drug-on” approach is reversible and tuneable, it requires chronic treatment with the Shld1 ligand to rescue the POI levels [23]. As such, this system provides a great strategy to analyse constitutively active enzymes, where Shld1-induced stabilization would trigger the experimental condition, rather than inhibiting it [23]. Despite the potential caveats of these drug-on methods, the applicability of this research is far reaching and has also formed the basis for subsequent attempts aimed at generating other effective proteolytic systems, such as the dTAG system for POI degradation which will be discussed later.

Another type of degron that can be stabilized upon the addition of a ligand has been engineered recently after the discovery of a new fluorescent protein from eel muscles. This protein, termed UnaG (*Unagi* eel green fluorescent protein), takes on a β-barrel shape which is capped by two α-helices. Fluorescence, with maximal absorption at 498 nm and maximal emission at 527 nm, requires bilirubin (BR) as a cofactor, placing it within the barrel structure of UnaG [33]. Random mutagenesis and screening of UnaG yielded a two amino acid substitution mutant (A36V, R136G), which acts as a destabilizing degron on fusion proteins in the absence of BR (tested on mCherry, proteasome interactors zfand2a and zfand2b, ubiquitin conjugating enzyme ube2n and the cell cycle regulator p21) (Fig. 2b) [22]. Notably, fusion of the UnaG degron to either terminus elicited degradation of the fusion proteins by the 26S proteasome, and this degradation could be halted by BR treatment [22]. Studies on the kinetics of UnaG-mediated degradation have shown that fusion proteins were completely degraded 4 h after removal of BR from the growth medium. Additionally, the system’s mechanism exhibited dose-dependent degradation of fusion proteins [22]. With a molecular weight of roughly 15 kDa, UnaG is only half the size of GFP, providing the advantage of a much smaller tag size when fused to target proteins, thereby reducing the potential unwanted effects that large tags can have on POIs.

## SMASh-tag degron

An alternative degron for controlled target protein degradation utilizes part of the hepatitis C virus (HCV) non-structural protein 3 (NS3) protease [34]. From the HCV transcript, one continuous protein is expressed, which is partially self-cleaved into the respective proteins by proteases, including NS3 [35]. This property of NS3 has been utilized for other molecular and cellular tools previously [36], where fusion of the NS3 protease domain to NS4 (a cofactor for NS3) acted as a destabilizing degron [34, 36]. The exact mechanism of how this degron works has not yet been elucidated. However, the authors surmised that deletion of the cleavage site, between the NS3 protease domain and NS4, induces the degron-like function, as NS4 needs a free N-terminus for ER membrane integration. With the terminus being blocked by NS3, NS4 presumably retains degron-like properties [34].

Fused to either the N- or C-terminus of a POI, and connected by an NS3 cleavage site, the degron continuously removes itself from the fusion protein, leaving the POI untagged within the cell (Fig. 2c). Upon treatment with specific protease inhibitors, like asunaprevir, which target the NS3 active site, the degron remains attached to the target protein leading to rapid POI degradation. Control over this system through the use of small molecules led to the name SMASh-tag (small molecule-assisted shutoff) [34]. Fusion of a SMASh-tag to either terminus of target proteins exhibits a degron-like function, and protein stability is tuneable with asunaprevir doses ranging from 0.15 nM (slightly reduced levels) to 1.5 µM (undetectable).

SMASh-tag provides an additional advantage on the study of protein half-life or degradation, as addition of asunaprevir only affects newly synthesized proteins and only those proteins tagged with the SMASh tag. Therefore, degradation of the previously generated pool of the SMASh-tagged target protein can be monitored without affecting any other proteins, which is not the case for conventional methods like cycloheximide treatment for ribosomal inhibition. No adverse effects on cell growth induced by asunaprevir were reported [34]. Additionally, in combination with CRISPR/Cas9 technology, this method might prove to be very powerful in studying essential proteins.

## TRIM away

**Fig. 3:**
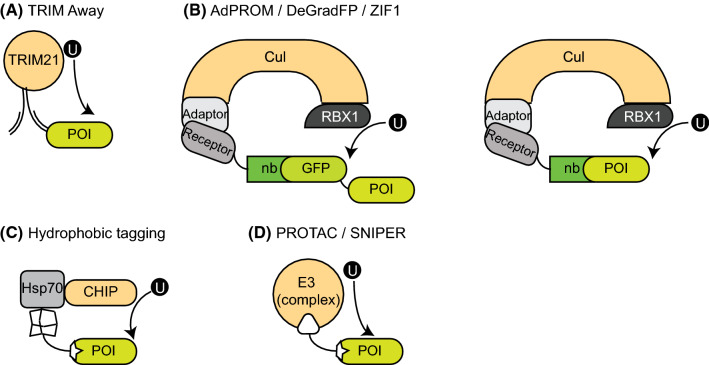
An overview of the targeted proteolytic approaches using high-affinity binders. **a** TRIM-away: the F_C_ region of POI-specific antibodies, once injected into cells, is recognized by TRIM21, while the F_ab_ binds the POI. TRIM21 marks the POI for degradation through ubiquitination. **b** AdPROM/DeGradFP/ZIF1: a cullin substrate receptor is fused to a target-specific or GFP-specific nanobody and expressed in cells. While the nanobody (nb) recognizes the POI/GFP, the receptor is recruited to the cullin–RBX–adaptor complex. Presentation of the POI to RBX1 results in ubiquitination and degradation of the POI. AdPROM and ZIF1 utilize the CUL2 system with EloB/C as adaptors, while employing VHL and ZIF1 as substrate receptors, respectively. DeGradFP works with the CUL1 system, using SKP1 as an adaptor, and Slmb F-box as the substrate receptor. **c** Hydrophobic tagging: a synthetic molecule with a POI-binding moiety binds to the target protein. The fused hydrophobic adamantyl group leads to ubiquitination and degradation of the POI, presumably through the recruitment of the molecular chaperone Hsp70 and its co-chaperone, the E3 ligase CHIP. **d** PROTAC/SNIPER: a synthetic molecule with two warheads interacts with an E3 ligase on one end and a protein of interest on the other. Spatial proximity allows for ubiquitination of the POI which is subsequently degraded

TRIM21 belongs to the family of tripartite motif (TRIM) proteins, which are involved in various cellular processes, including antiviral responses [37]. TRIM proteins consist of a RING-box, which mediates the E3 ligase activity, a B-box and a coiled-coil domain [38]. Additionally, TRIM proteins contain a C-terminal PRYSPRY domain, which is thought to confer target specificity for the different TRIM members [38]. The TRIM-21 PRYSPRY domain interacts with the constant F_c_ region of IgG antibodies with affinity in the low nanomolar range, with a TRIM21:F_c_ ratio of 2:1 [38]. Uncharacteristically for F_c_ receptors, TRIM21 is localized in the cytosol, which in part explains its function in conferring intracellular immunity against viruses. Antibodies bound to viruses are co-endocytosed, leading to recognition by TRIM21, and subsequent autoubiquitination and degradation of the virus–antibody–TRIM21 complex [39]. The Schuh research group developed a targeted proteolytic system, called TRIM-away, by exploiting this unique function of TRIM21. Following microinjection of a target-specific antibody, the POI is recognized by TRIM21, ubiquitinated and rapidly degraded (Fig. 3a) [40]. Using a GFP-specific antibody for microinjection into TRIM21 expressing cells, GFP levels were depleted with an apparent half-life of roughly 16 min [40]. The system could also be applied to primary cells, in which achieving protein silencing with RNAi or CRISPR/Cas9 is difficult. TRIM21 was able to target all accessible cellular proteins, although for nuclear-localized proteins, a target-specific nanobody has to be fused to F_c_ fragments due to the full-length antibody being too large to pass through the nuclear pore complex. As shown by targeting huntingtin, TRIM-Away was also able to distinguish between wild-type and mutant proteins, depending on the specificity of the delivered antibody [40].

The power of TRIM-away was also shown by its ability to target and degrade Rec8 in mouse oocytes within minutes [40]. Rec8 is a meiosis-specific component of the cohesion complex, required for tethering of sister chromatids in oocytes throughout the entire meiotic process. As such, Rec8 does not turnover and is thought to have a half-life of several years in humans [7]. RNAi approaches lack the persistence to affect Rec8 protein levels, while CRISPR is not a viable option due to Rec8 being an essential gene.

TRIM-away was also able to impact signalling pathways downstream of target protein degradation. The longevity of TRIM-away efficacy was assessed to be roughly 3–4 days. However, this was dependent on the levels of target protein within cells, the amount of antibody being applied and the amount of TRIM21 within cells. To widen the applicability of the system beyond single cell analysis, the authors developed a bulk cell approach by using electroporation, showing specific effects on signalling pathways like ERK1 or mTOR [40]. This approach additionally allows for co-electroporation of TRIM21, omitting the stable overexpression lines needed previously. Alternatively, cell lines inherently expressing high amounts of TRIM21 can be used without co-electroporation or microinjection of TRIM21, facilitating the use of the system [40]. However, getting large amounts of pure and highly selective IgG antibodies into target cells is not only challenging, but extremely costly and therefore makes TRIM-away not as feasible as other proteolytic methods, especially when considering large-scale applications.

## Affinity-directed PROtein Missile system (AdPROM)

Recent data from our own laboratory described a novel and efficient proteolytic system that combines the endogenous cullin 2 (CUL2) CRL machinery, with high-affinity polypeptide binders for target protein recognition [18, 19]. We called this approach the Affinity-directed PROtein Missile (AdPROM) system. AdPROM works by first expressing the Von Hippel Lindau (VHL) protein, the substrate receptor for CUL2 [41], fused to a polypeptide binder that recognizes a particular POI, in cells (Fig. 3b). We have used both camelid-derived VHH domain nanobodies and synthetic binders that use the human fibronectin type III domain as a backbone—so-called monobodies [42]—for this purpose. Such small polypeptide binders are ideal for intracellular expression, as they do not require complex folding or disulphide bridge formation, unlike conventional antibodies. Upon successful expression in cells, the VHL–nanobody/monobody fusion protein binds the protein of interest and recruits it to the endogenous CUL2 machinery through the association of VHL with the elongin B and C adaptor proteins [41, 43, 44]. Every cullin E3 ligase complex possesses a substrate receptor such as VHL, adaptor protein(s), a cullin and an RBX E3 ligase. Once the substrate receptor binds and recruits the substrate to the cullin complex, the substrate is in a prime position for RBX1/2-mediated ubiquitination [44, 45]. Cullins themselves are regulated by the post-translational modification of NEDDylation, which serves to attach the small ubiquitin-like modifier NEDD8 onto lysine residues of the cullin, resulting in conformational changes required for optimal substrate ubiquitination [43, 46, 47]. With regard to the AdPROM system, the target POI, once recruited to the CUL2 complex, is ubiquitinated by RBX1 and marked for destruction through the proteasome [18, 19].

By fusing VHL with the VHH domain nanobodies recognizing the inflammasomal protein ASC [48], or by fusing VHL to either of the two monobodies that specifically bind the tyrosine phosphatase SHP2 [42], we showed robust degradation of endogenous ASC and SHP2 proteins, respectively, in a whole host of human cancer cell lines [19]. These findings demonstrate that if a POI-targeting nanobody or polypeptide binder is available, the AdPROM system can be applied in any cell line to target POI degradation rapidly. Alternatively, for POIs for which the binding nanobodies/monobodies do not yet exist, we generated GFP-tagged knockin POIs through CRISPR/Cas9 gene editing technology, and utilized nanobodies recognizing GFP fused with VHL to induce degradation of endogenous GFP-tagged PAWS1 and VPS34 [18]. Excitingly, targeted degradation of GFP-VPS34 also led to the degradation of endogenous UVRAG, a protein which forms a stable complex with VPS34 [18]. As expected, the degradation of VPS34 by AdPROM resulted in reduction in levels of endosomal phosphatidylinositol (3,4,5)-trisphosphate [18]. The targeted proteolysis by AdPROM is dependent on both proteasomal activity and cullin NEDDylation [18, 19]. Crucially, despite VHL being the substrate adaptor for endogenous hypoxia-inducible factor 1α (HIF1α) [49], expression of AdPROM does not appear to interfere with hypoxia signalling, as evidenced by no detectable stabilization of HIF1α in AdPROM-expressing cells [18, 19]. That being said, there may be other endogenous VHL targets and functions that have not yet been identified, and the impact of the AdPROM system on these and downstream biology needs to be tested further. AdPROM has not yet been applied for POI degradation in whole organisms.

## DeGradFP and ZIF1 proteolytic systems

Similar to AdPROM, other E3 ligase systems have been combined with nanobodies recognizing GFP (GFPnb) to induce degradation of endogenously GFP-tagged POIs in intact organisms. One such approach, named deGradFP, utilized a fusion of an anti-GFP nanobody with the F-box domain from the *Drosophila melanogaster* Slmb protein, which is a substrate receptor component of the SCF E3 ligase complex, and demonstrated efficient degradation of GFP-tagged proteins in *D. melanogaster* (Fig. 3b) [50, 51]. Similarly, another system harnesses the *Caenorhabditis elegans* SOCS/CUL2 CRL complex for targeted degradation of proteins. The *C. elegans* SOCS-Box protein ZIF1 recruits proteins containing a 36 amino acid ZF1 motif and targets them for degradation [52]. By tagging POIs with such a ZF1 motif, it is possible to recruit and degrade them via endogenous CRL machinery [52]. However, some disadvantages with this system are that ZIF1 itself is regulated in different stages of development [53] and POI degradation will compete with endogenous substrates. More recently, the ZIF1 system was also adapted to create a deGradFP system [54]. The ZIF1 protein was fused to a GFP nanobody (GFPnb) and expressed in *C. elegans* [50]. In combination with CRISPR/Cas9 gene editing technology, it was demonstrated that the ZIF1–GFPnb polypeptide recruits and degrades GFP-tagged knockin POIs in vivo (Fig. 3b) [50]. These approaches showcase the ability to degrade GFP-tagged POIs in an intact organism, and provide further merit for the applicability of nanobody-based high-affinity binders in proteolytic biotechnology.

## Hydrophobic tagging (HyT)

Cells have developed an elaborate surveillance mechanism to help and maintain proteins in their proper folding state. Unfolded proteins are bound by molecular chaperones, which prevent their aggregation and help them regain their original folding [55]. However, molecular chaperones Hsp70 and Hsp90 can also recruit the co-chaperone E3 ligase CHIP (C-terminus of Hsp70 interacting protein), which leads to ubiquitination and degradation of the chaperone-bound client proteins [56–59]. To utilize this surveillance system for targeted proteolysis, the Crews lab created a small molecule with a POI-binding moiety coupled to a hydrophobic group for permanent chaperone binding (Fig. 3c). As a proof of principle, a HALO-tag reactive linker was joined with a hydrophobic adamantyl group. Treatment of cells expressing GFP-HALO with this compound (HyT13) resulted in efficient degradation (50% in 8 h at 1 µM), without notable toxicity even at concentrations up to 20 µM [56]. Remarkably, HALO-tagged fusions of transmembrane proteins with differing amounts of membrane spanning helices could be degraded by this system as well [56].

In an attempt to make the system more applicable and remove the need to create HALO-fusions, the HALO reactive linker was exchanged for the Alzheimer’s disease-related protein Tau-interacting peptide (the C-terminal region of tubulin). This induced efficient degradation of Tau [60]. A similar result was achieved when the HALO reactive linker was switched with a high-affinity agonist for the androgen receptor, RU59063 [57], or with a newly developed inhibitor against the EGFR tyrosine kinase HER3, TX1-85-1 [61]. In all three cases, the high-affinity binders were linked to the hydrophobic adamantyl group. However, an exact mechanistic explanation regarding this system, and any off-target effects resulting from potential perturbations of the unfolded protein response pathways, remains to be delineated.

## PROteolysis-TArgeting Chimeras (PROTACs)

Unlike most of the POI degradation systems described thus far, PROteolysis-TArgeting Chimeras (PROTACs) against endogenous POIs do not rely on prior genetic modification of the target gene. They are small bivalent molecules that simultaneously bind to the POI and an E3 ligase component, ultimately leading to POI ubiquitination and degradation (Fig. 3d). The first PROTAC described consisted of an ovalicin headgroup that bound methionine-aminopeptidase 2 (MetAP-2) linked to an IκBα derived peptide headgroup that bound the Skp1–cullin–F-Box (SCF) complex [62]. This molecule efficiently degraded MetAP-2 in *Xenopus* oocyte extracts, however it was not cell permeable [62]. Since then great efforts have gone into developing PROTACs that can traverse the cell membrane and target different E3-ligases and target proteins. Some of these PROTACs will be explored below.

## MDM2-based PROTACs

Mouse double minute 2 homolog (MDM2) is an oncoprotein with E3 ligase activity that targets the tumour suppressor p53 for ubiquitination. MDM2 binds the p53 transactivation domain, leading to the inhibition of p53 function, nuclear export and degradation under physiological conditions. Upon cell stress, this interaction is disrupted leading to increased levels of active, nuclear-localized p53. However, MDM2 is one of the most upregulated E3 ligases in cancer, leading to constant removal of p53 [63–65]. To utilize MDM2 for the PROTAC system, the discovery of nutlins, small molecules that bind MDM2 at the p53 interaction interface, was a crucial step [66]. In an initial PROTAC approach, the nutlin headgroup was combined with a selective androgen receptor modulator (SARM) moiety, and androgen receptor degradation was observed in a transient overexpression system [67]. Recently, a potent PROTAC has been developed that connects the MDM2-binder idasanutlin and the bromodomain containing protein 4 (BRD4) inhibitor JQ1. This PROTAC, A1874, degraded BRD4 almost completely in HCT116 cells at 100 nM within 24 h. Additionally, PROTAC treatment led to the stabilization of p53 and had an antiproliferative effect that was synergistic when compared to treatments with either inhibitor alone [68].

## Cereblon (CRBN)-based PROTACs

CRBN is a substrate recognition unit of the CUL4–RBX1–DDB1 E3 ligase complex [69]. CRBN was shown to bind the drug thalidomide in its substrate binding pocket, thereby creating a new surface to recruit other proteins for ubiquitination and degradation, such as the transcription factors Ikaros and Aiolos (IKZF1 and 3, respectively) or the kinase CK1α [70–72]. However, thalidomide displaces endogenous CRBN substrates, such as the transcription factor MEIS2 which is involved in embryonic development, perhaps explaining its teratogenic effects in pregnant women [69, 73–75]. Nevertheless, thalidomide and its derivatives, e.g. lenalidomide and pomalidomide, have been used for a newfound purpose in their immunomodulatory imide drug (IMiD) function and are applied in the treatment of several diseases, including erythema nodosum leprosum (ENL), multiple myeloma, myelodysplastic syndrome (MDS), systemic lupus erythematosus and inflammatory bowel disease [75, 76].

The compound CC-885 was recently added to the available pool of CRBN modulators. While maintaining the binding and degradation capability for IKZF1, CC-885 targeted GSPT1, a translation termination factor, for degradation. Notably, other available IMiDs did not have an effect on GSPT1 protein stability [77]. Structural analysis revealed the minimal requirement for IMiD targets to participate in the CRBN–IMiD interaction to be a surface-exposed turn, containing a precisely positioned glycine residue [77, 78]. Additional interactions occur via hydrogen bonding of the surrounding backbone. However, target-specific docking sites can be presented by the thalidomide derivative, as is the case with CC-885 and GSPT1 [77], as well as pomalidomide and the zinc finger proteins IKZF1 or ZNF692 [78]. A proteomic approach has also shown that a large subset of zinc finger proteins, in particular those containing Cys_2_-His_2_ (C2H2) zinc finger domains that exhibit the specific glycine containing turn structure, can be degraded by IMiD-bound CRBN. Additionally, different IMiDs have been shown to induce the degradation of distinct Zn-finger proteins, which is attributed to steric and structural properties of the targets, and the CRBN–IMiD surface [78]. As such, the CRBN–IMiD-target interaction is not dictated by a specified sequence, but rather a structural motif.

While IMiDs undoubtedly provide a useful therapeutic approach for different diseases, their use in the context of targeted protein degradation is currently limited, as they would have to be painstakingly optimized. Nevertheless, thalidomide and its derivatives provide a valuable warhead for the development of CRBN targeting PROTACs. Recently, the anaplastic lymphoma kinase (ALK) was degraded by two PROTACs based on a pomalidomide warhead targeting CRBN linked with either LDK378 (ceritinib) [79, 80] or TAE684 [79] targeting ALK. Both PROTACs were able to reduce downstream signalling and proliferation of ALK-driven cell lines by degrading ALK [79, 80]. However, both PROTACs retained their inherent off-target binding of Aurora kinase A, as it too was degraded [79].

Another example of efficient utilization of CRBN for protein degradation was achieved with BRD4. The scaffold protein for transcriptional elongation factor P-TEFb [81] preferentially binds upstream of several oncogenes such as c-myc as so-called super-enhancers [82]. While protein–protein interactions of BRD4 can be inhibited by OTX015 or JQ1, which lead to suppressed levels of c-myc, the inhibitory effect is quickly alleviated once the inhibitor is removed from cells [83, 84]. PROTACs consisting of pomalidomide and OTX015 [84] or JQ1 [85] resulted in robust BRD4 degradation when used at low nanomolar concentrations [84, 85]. Concomitantly, c-Myc levels were strongly depleted, and this depletion lasted longer after washout of the PROTAC than after inhibitor treatment [84].

An important point to keep in mind when working on targeted protein degradation is the compatibility of the desired target protein to be degraded, and the E3 ligase to be used, as exemplified by another PROTAC target of CRBN. In a dual approach, tyrosine kinase inhibitors targeting the oncogenic fusion protein BCR–Abl, i.e. bosutinib or dasatinib, were either incorporated into a PROTAC targeting CRBN, or VHL. While binding of the PROTACs to BCR–Abl still inhibited downstream signalling, protein levels remained unchanged with any of the tested VHL-PROTACs. Interestingly, c-Abl could be degraded with a dasatinib–VHL PROTAC at 1 µM, however, higher concentrations left c-Abl protein levels unaffected [86]. This is likely due to the so-called ‘hook-effect’, where high concentrations of the PROTACs block ternary complex formation, with either component bound to individual PROTAC molecules. In contrast, both bosutinib- and dasatinib–pomalidomide PROTACs were able to efficiently degrade both BCR–Abl and c-Abl at around 100 nM concentrations and the dasatinib–pomalidomide PROTAC was efficient at reducing cell viability with a 1000-fold higher efficiency over non-BCR–Abl driven cell lines [86].

PROTACs can also be utilized to degrade E3 ligases. To counteract the negative effects MDM2 has on cancer progression, the inhibitor MI-1242, which binds MDM2 with low nanomolar affinities, was coupled to lenalidomide. Linker adjustments led to two very potent PROTACs, MD-222 and MD-224. These PROTACs are very efficient at degrading MDM2 and, in turn, stabilize p53 and inhibit cell proliferation at low nanomolar doses. Similar to BCR–Abl, this could not be achieved by the use of a VHL warhead [87].

## VHL

One of the most studied E3 ligase adaptors for PROTAC development is the VHL tumour suppressor. As described in the AdPROM section, VHL acts as the substrate receptor for the Cul2-Rbx1 E3 ligase complex, recruiting HIF1α for its ubiquitination and degradation [88]. Under normoxic conditions, the VHL–HIF1α interaction is mediated through a hydroxyproline residue on HIF1α [49]. This hydroxyproline residue was the starting point for a peptidic ligand molecule with an IC_50_ value of 4.1 µM for VHL binding at the HIF1α binding site [89], which could be improved to sub-micromolar affinity [90]. Since peptidic molecules provide a challenge in terms of cell permeability of compounds, the peptidic VHL-binding portion was substituted subsequently by a small molecule, which retained the critical hydroxyproline [20]. Using a warhead targeting either the estrogen-related receptor alpha (ERRα) [91] or the serine threonine kinase RIPK2, efficient degradation of both proteins could be achieved with doses ranging from 3 nM (RIPK2) to 100 nM (ERRα) [20]. Similar positive outcomes could be achieved with PROTACs targeting TANK-binding kinase 1 (TBK1) [92], and ALK, where a clear correlation between efficacy in inhibiting cell proliferation and ALK driver status in different cell lines could be observed [93]. BRD4, which was efficiently degraded with a CRBN PROTAC [84], could also be targeted using a VHL-recruiting warhead. However, in this case, the BRD inhibitor JQ1 was chosen. While the PROTAC exhibited a clear preference for BRD4, BRD2 and 3 were also degraded at either high concentrations or prolonged treatments [94].

More recently, homo-PROTACs for VHL have been developed. These molecules harbour two VHL binding moieties, rendering VHL both the recruiter as well as the ubiquitination substrate [95]. The most active compound, CM11, specifically depleted the long isoform of VHL, VHL30, within 4 h at 10 nM concentration, providing an intriguing tool for research on isoform specific functions of VHL.

## c-IAP PROTACs and specific and non-genetic IAP-dependent protein erasers (SNIPERs)

Cellular inhibitor of apoptosis 1 and 2 (c-IAP1 an c-IAP2) proteins were initially identified as inhibitors of caspase 3 and 7, which bind caspases through their baculovirus IAP repeat (BIR) domains [96]. c-IAP proteins were later shown to regulate components of the NF-κB signalling pathway through their RING domain catalytic activity [97]. A decade ago, methyl-bestatin (ME-BS) was identified as a potent binder of c-IAP1, which induced autoubiquitination and degradation of c-IAP1 by binding its BIR3 domain [98]. In an initial PROTAC approach for c-IAP, ME-BS was coupled to all-trans retinoic acid (ATRA), which binds cellular retinoic acid binding protein (CRABP-1 and -2), with the latter chosen due to the involvement of CRABP1/2 in Alzheimer’s disease, neuroblastoma, Wilms tumor, and head and neck squamous cell carcinoma [99]. PROTAC treatment of cells expressing c-IAP1 and either CRABP-1 or CRABP-2 reduced levels of both c-IAP1 and either CRABP protein. Using the PROTAC on neuroblastoma, degradation of CRABP-2 resulted in reduced cell migration [99]. In an attempt to improve both the stability and affinity of the compound, the ME-BS moiety was replaced with MV1, a potent binder of c-IAP1, c-IAP2 and XIAP [100]. Treatment of cells with this new PROTAC also induced efficient degradation of both c-IAP1 and CRABP-2 and blocked proliferation of neuroblastoma cells; however, the effects on either c-IAP2 or XIAP were not analysed [100].

A subgroup of c-IAP PROTACs are commonly referred to as ‘specific and non-genetic IAP-dependent protein erasers’ (SNIPERs) [101, 102]. To date, SNIPERs that target CRABP-2 [101], estrogen receptor alpha (ERα) [103], and transforming acidic coiled-coil-3 (TACC3) [104] have been generated, and in each case, successful degradation of the target protein has been demonstrated. To improve on the cIAP1 ligand-binding affinity, the IAP antagonist LCL161 was chosen as an alternative cIAP1-binding moiety to create a novel SNIPER. Using this new approach, the authors demonstrated robust degradation of ERα, the chronic myeloid leukemia (CML)-causative BCR–Abl fusion protein, BRD4 and PDE4 proteins in cells [102].

## Keap1-based PROTACs

Recently, a peptide PROTAC was developed that was able to tether Kelch-like ECH-associated protein-1 (Keap1) and Tau into a ternary complex [105]. Keap1 functions as a substrate receptor unit for the cullin3–RBX1 complex, targeting nuclear factor erythroid 2 related factor 2 (Nrf2) for ubiquitination under physiological conditions, while oxidative stress inactivates Keap1, leading to accumulation of active Nrf2 [106]. A peptide inhibitor of the Nrf2–Keap1 interaction had been developed previously, with an apparent K_D_ of 2.8 nM for Keap1 binding [107]. To develop a peptide PROTAC to recruit the microtubule associated protein Tau to Keap1, this peptide was fused to a short peptide sequence of β-tubulin (YQQYQDATADEQG), which in turn was fused to a C-terminal poly-d-arginine sequence for enhanced cell permeability of the peptide [105]. Generation of the full-length peptide decreased Keap1 affinity tenfold and exhibited affinity towards Tau in the sub-micromolar range. This PROTAC, termed peptide 1, was able to degrade overexpressed GFP-tagged Tau in different cell lines at 20 µM after 6–12 h of treatment times to various degrees.

## HALO and dTAG PROTACs

As PROTACs require highly selective small molecule binders of POIs, currently they have limited utility against the vast majority of endogenous proteins. Developing selective high-affinity binders of POIs is both resource- and time-intensive process. Therefore, moieties that recognize polypeptide tags, such as HALO and dTAG that could be inserted into POIs by CRISPR/Cas9 genome editing, offer opportunities for inducible degradation of POIs by using the tag-specific PROTACs (such as HaloPROTACs and dTAG-13, respectively) [108, 109]. HaloPROTACs, which bind to the HALO tag on POIs on one side and VHL on the other, have been successfully employed to rapidly and inducibly degrade HALO-tagged POIs through the VHL/CUL2/RBX1-dependent UPS [109]. However, one of the limitations of using the HALO tag is its apparent large size (~ 33 kDa). This has the potential of affecting the function of the POI by obstructing key interactors, or substrates from accessing the POI, and in certain cases, the HALO tag itself might become the primary target of ubiquitination and degradation.

To overcome this, a smaller polypeptide tag that also harnesses a high-affinity PROTAC-binding moiety is desirable. Excitingly, Nabet et al. recently described such a system using an FKBP12(F36V) mutant (dTAG; ~ 12 kDa) [108]. The authors exploited an FKBP12-F36V-directed ligand called AP1867 (Ariad Pharmaceuticals) to generate a heterobifunctional PROTAC dTAG-13 that recruits dTAG to CRBN, and thereby degrades dTAG-fused POIs [108]. This approach proved successful in degrading dTAG–BRD4 fusion proteins generated by CRISPR/Cas9 gene editing, and showed selective, rapid degradation of dTAG–BRD4 upon ligand treatment [108]. The authors extended this analysis and showcased consistent utility in degrading dTAG–KRAS–G12V and dTAG–EZH2 proteins overexpressed in mammalian cells. However, the authors noted varying rates of POI degradation, depending on the POI used, possibly owing to limited accessibility of the dTAG ligand in different subcellular compartments [108]. The efficacy of dTAG-13 to degrade overexpressed dTAG–KRAS–G12V was shown in mice, thereby establishing the dTAG system as an elegant FKBP12-based proteolytic system [108].

## Future perspectives

The field of targeted proteolysis has taken huge strides in a relatively short period of time, both in terms of its development as a robust research tool, and its potential in therapeutic applications. While cell-permeable and selective small molecule POI degraders, such as PROTACs and SNIPERs, remain the ultimate choice in both research and therapeutics, the availability of other degron and nanobody-based targeted proteolytic tools offer more rapid opportunities to achieve targeted POI degradation. Developing small molecule PROTACs, SNIPERs and IMiDs to degrade specific POIs remains extremely challenging, as the generation of high-affinity and selective POI-binding ligands that can be fused with the E3-binding ligands is costly and time-consuming. As a consequence, any rapid, targeted proteolytic approaches that can achieve the desired loss-of-function phenotypes have the potential to streamline POIs for the development of costly cell-permeable proteolytic small molecules, such as PROTACs. In this context, AiD and nanobody-based degradation systems such as AdPROM offer much promise.

The premise for targeted proteolysis lies in harnessing the cellular ubiquitination machinery and the proteolytic pathways. Although there are over 600 E3 ubiquitin ligases encoded by the human genome, this review has demonstrated that only a handful of E3 ubiquitin ligases have been utilized by the current targeted proteolytic methods. Increasing the portfolio of E3 ubiquitin ligases that promote efficient proteolysis is desirable to enhance the capabilities of targeted proteolytic technologies. In this context, nanobody-based technologies such as AdPROM can rapidly inform whether a specific E3 ligase is a suitable candidate [19]. For any targeted proteolytic system to work effectively, the POI when recruited to the E3 ligase component has to present lysine (or other) residues for ubiquitination. In cases where the POI is recruited to the E3 ligase component, but is not degraded, absence of POI ubiquitination might render the proteolytic system ineffective. For some proteins, it might transpire that the UPS is not able to cause proteolysis of a POI despite its ubiquitination. All of the current targeted proteolytic systems utilize the UPS to achieve POI degradation. However, harnessing ubiquitin-mediated lysosomal degradation of POIs could also offer an expansion to the targeted proteolytic toolkit.

Most of the targeted proteolytic systems that require tagging of the POI with a polypeptide tag, such as GFP or dTAG, using CRISPR/Cas9 are useful for understanding POI function, but are limited in scope to the cell systems in which CRISPR/Cas9 modifications are feasible. Moreover, tagging proteins can compromise the structural integrity, subcellular distribution and biological activity of the POI itself. Ideally, any system that can target endogenous, unmodified POIs for degradation is desirable and enables research on the POI function in any cell line. Other than PROTACs, the AdPROM system offers an ideal toolkit to rapidly degrade endogenous POIs, provided there are selective POI-targeting polypeptides. These POI-targeting polypeptides can either be single chain antibodies (e.g. variable domains from camelid or shark-derived heavy chain only antibodies, nanobodies), or a variety of synthetic polypeptide scaffolds that bind POIs such as fibronectin type III domain-based monobodies [110, 111], designed ankyrin repeats (DARPINs) [112, 113], cystatin-derived affimers [114] or Affibodies derived from the Z-domain of staphylococcal protein A [115]. These small polypeptide scaffolds consist of a stable backbone and variable loop regions, which determine POI-binding specificity. The mutagenized variants are screened for their interaction with specific POIs, typically through phage display technologies [116].

Recent and ongoing advances have steadily increased the repertoire of small polypeptide high-affinity protein binders of different protein targets (Table 1) and more are likely to follow. While high-affinity binders are already used for immunoprecipitation and immunofluorescence applications [117], their use with AdPROM and AdPROM-like technologies will vastly expand the toolkit for researchers. For any polypeptide POI-binder to be an effective degrader when incorporated into the AdPROM system, it has to bind the POI in cellulo. As such, AdPROM currently only works on intracellular targets. The templates for developing these high-affinity binders are bacterially expressed recombinant proteins, which allow for detection of unmodified targets. With more technological advances, designing small polypeptide binders that recognize particular conformations of the POI (e.g. active or phosphorylated protein states) may allow for targeted destruction of distinct subsets of the POI, to enable highly specific loss-of-function assays to be performed, without interference from the non-relevant POI pools.

**Table 1:** Compilation of currently available high-affinity polypeptide binders of intracellular proteins

POI binder	Targets
Nanobody	GPCR Gβ_1_γ_1_ [122], C-Myc [123]; LMO2 [124]; endothelial and epithelial kinase (Etk) [125]; protein kinase Cε [126]; CapG [127]; RAS [128]; caspase-3 [129]; Bax [130]; hnRNP-K [131]; L-plastin [132–134]; fascin [135]; cortactin [135]; gelsolin [136]; huntington [137, 138]; α-synuclein [139]; β-catenin [140–142]; nuclear poly(A)-binding protein 1 (PABPN1) [143]; GFP [144], ASC [48]
Monobody	β-Catenin [145]; Abl (SH2 domain) [111, 146]; SUMO1 [111, 147, 148]; Aurora A [149]; WDR5 [150]; H/K-RAS [151]; GFP [111]; Erk-2 [152]; ubiquitin [110]
DARPIN	GFP [153]; tubulin [154]; caspase-2 [155, 156]; caspase-7 [155, 156]; H/K/N-RAS [157]
Affimer/Affibody	p85α [158]; GRB2 [158]; GRB7 [158]; GRB10 [158]; GRB14 [158]; F-actin [159]; SUMO1 [160]; SUMO2 [160]; SUMO1/2 [160]; c-JUN [161]; amyloid-beta [162, 163]

The list presents a compilation of some of the reported nanobodies, monobodies, DARPins and Affimers which might be suitable for use as POI-targeting elements for AdPROM and AdPROM-like technologies. Note that only nanobodies for intracellular proteins/epitopes are listed in this table and appropriate references are cited

For many proteins known to be involved in driving diseases, such as KRAS in many cancers, conventional drug development strategies often aimed at developing inhibitors have failed [118, 119]. In drug research, such proteins are labelled as “undruggable” targets. Targeted protein degradation offers a fresh and potentially viable approach at drugging these targets. Indeed, some PROTACs, such as the androgen receptor degrader PROTAC, ARV-110, has shown pre-clinical efficacy against prostate cancer [120] and is now a candidate for clinical trials [121]. Even in the absence of robust PROTAC molecules to target a wide range of so-called undruggable POIs, nanobody-based targeted proteolytic systems, such as AdPROM, can rapidly address the druggability of any given target in different disease models. Given the availability of multiple toolkits for targeted proteolysis, their utility in drug discovery research is undoubtable, and the coming years are sure to witness major advances.
